# Implementation of an infection prevention framework in a psychiatric hospital: Processes from two Mental Health centers

**DOI:** 10.1192/j.eurpsy.2026.11019

**Published:** 2026-07-31

**Authors:** R. Kigli, M. Ben Tzarfati

**Affiliations:** 1 Nursing, Tel Aviv University, Tel Aviv; 2 management, Merhavim Mental Health Center, Be’er Yakov; 3 Nursing, Abarbnel Mental Health Center, Bat Yam, Israel

## Abstract

**Introduction:**

In recent years, infection prevention and epidemiology have gained prominence within healthcare safety and quality frameworks, particularly in mental health settings. Recognizing the need for healthcare systems to provide both therapeutic care and a safe physical, emotional, and bacteriological environment, Israel’s Ministry of Health has established guidelines covering staff onboarding, required vaccinations, disinfection protocols in inpatient and specialized units, handling hazardous materials, preventing infections in contagious wards, and tailored measures for specific populations.

**Objectives:**

To develop and implement a comprehensive infection prevention framework in a psychiatric hospital, aligning with Ministry of Health regulations and advanced professional standards at organizational, departmental, and therapeutic levels.

**Methods:**

The implementation process included:

Appointing a certified “Epidemiology and Infection Prevention Coordinator”.

Establishing an “Infection Prevention Committee” - developing and updating professional protocols and guidelines.

Training departmental quality assurance representatives- enforce guidelines, conduct audits, and provide patient and staff training.

Implementing seasonal and periodic vaccination campaigns based on identified needs.

Participating in Ministry of Health audits.

Issuing periodic reports as per regulatory requirements.

Conducting organization-wide training and knowledge refreshers.

**Results:**

Implementing the framework required collaborative teamwork and prioritization, particularly during a prolonged war period that impacted staff availability, elevated stress levels, and strained resources across diverse units (e.g., pediatric, psychogeriatric, forensic, acute, and long-term care). Unique challenges arose in managing COVID-19 in closed acute units, necessitating integration of mental health treatment with specialized infection prevention measures to protect patients and multidisciplinary teams. The process was applied in two major mental health centers, addressing challenges in team coordination, inter-professional interfaces, consultations with Ministry of Health and regional health authorities, and departmental implementation.

**Conclusions:**

This presentation will outline the implementation process, challenges encountered, and solutions developed in two large mental health centers. It will discuss inter-professional collaboration, consultation processes with health authorities, departmental implementation strategies, and future plans for sustained organizational infection prevention efforts. The framework offers a model for balancing mental health care with robust infection control, particularly in high-pressure and resource-constrained environments.

**Disclosure of Interest:**

None Declared

